# Dissociating Object Directed and Non-Object Directed Action in the Human Mirror System; Implications for Theories of Motor Simulation

**DOI:** 10.1371/journal.pone.0032517

**Published:** 2012-04-10

**Authors:** Zarinah K. Agnew, Richard J. S. Wise, Robert Leech

**Affiliations:** 1 Institute of Cognitive Neuroscience, University College London, London, United Kingdom; 2 Cognitive Neuroimaging Group, Medical Research Council, Clinical Sciences Centre, Imperial College London, Hammersmith Hospital Campus, London, United Kingdom; 3 Division of Neuroscience and Mental Health, Imperial College London, Hammersmith Hospital Campus, London, United Kingdom; French National Centre for Scientific Research, France

## Abstract

Mirror neurons are single cells found in macaque premotor and parietal cortices that are active during action execution and observation. In non-human primates, mirror neurons have only been found in relation to object-directed movements or communicative gestures, as non-object directed actions of the upper limb are not well characterized in non-human primates. Mirror neurons provide important evidence for motor simulation theories of cognition, sometimes referred to as the direct matching hypothesis, which propose that observed actions are mapped onto associated motor schemata in a direct and automatic manner. This study, for the first time, directly compares mirror responses, defined as the overlap between action execution and observation, during object directed and meaningless non-object directed actions. We present functional MRI data that demonstrate a clear dissociation between object directed and non-object directed actions within the human mirror system. A premotor and parietal network was preferentially active during object directed actions, whether observed or executed. Moreover, we report spatially correlated activity across multiple voxels for observation and execution of an object directed action. In contrast to predictions made by motor simulation theory, no similar activity was observed for non-object directed actions. These data demonstrate that object directed and meaningless non-object directed actions are subserved by different neuronal networks and that the human mirror response is significantly greater for object directed actions. These data have important implications for understanding the human mirror system and for simulation theories of motor cognition. Subsequent theories of motor simulation must account for these differences, possibly by acknowledging the role of experience in modulating the mirror response.

## Introduction

### Distinguishing between object and non-object directed action

Praxis, the performance of skilled voluntary action, can be widely divided into actions involving objects, and actions not involving objects. It is well established that these different types of action are subserved by distinct neural networks [1], [2]. Neuroimaging has demonstrated differences in neural activity associated with perception of object directed and non-object directed action. For example, visual recognition of gestures compared to pantomimed object directed action has been associated with increased activity in the left inferior frontal gyrus [3]. Similarly, increased activity in inferior parietal and frontal regions has been reported for perception of meaningful compared to meaningless actions [4]. In relation to praxis, tool use is associated with activity in intraparietal sulcus and premotor areas (including F5) in the macaque brain, compared to object manipulation [5], [6] but relatively little is known about non-object directed movement of the upper limb in non0human primates. In the human, Culham et al. [7] have demonstrated a neural dissociation between execution of object directed and non-object directed movements. However, the most compelling evidence for a neural dissocation between object and non-object direct action comes from the clinical literature. Patients with ideational apraxia suffer from deficits in the performance of actions made on objects, in the absence of object agnosia. In contrast patients with ideomotor apraxia display selective deficits in the execution of non-object directed actions, such as gestures or sequencing movements in space and time [8]. These deficits to action/gesture production are typically associated with lesions in frontal and parietal regions, more so in the left hemisphere [1] and have been linked to deficits in action/gesture perception in a subset of patients [9], [10].

### Mirror neurones in the macaque and human brain

Mirror neurones are a subclass of motor neurone discovered in macaque premotor [11] and parietal cortex [12], [13], which are known to dissociate between the two kinds of actions such that they respond during observation and execution of object-directed actions, but not during non-object directed movement [12], [14]. Mirror neurones, visual and motor cells, that are active for non-object directed communicative mouth actions, such as lip smacking, have been found [15]. However, macaque monkey hand mirror neurons have only been investigated in relation to object-directed actions [12], [13], [14]. These cells, are also thought to exist in the human brain, [16], [17]. Whilst non-invasive techniques can only resolve large scale neural assemblies, it is largely accepted that a possible human mirror *system*, not mirror neurons [18] exists in premotor and parietal areas [19]. For example, perception of action has been associated with activity in cortical regions outside of the typical visual processing regions of occipital cortices such as parietal and premotor cortices [19], and broadly comparable patterns of activation within the premotor and parietal circuits have been reported during perception *and* execution of object directed and communicative action/gesture [20], [21], [22]. However, these studies only address either perception or praxis, not both and thus only speak indirectly to responses of the human mirror system.

### Implications for theories of motor cognition

Presently it remains unclear how the typical human mirror response, defined as common encoding of activity during perception and execution of object-directed (transitive) action, [14], compares to that of a non-symbolic/gestural movement. This issue has implications for theories of mirror neuron function; the existence of mirror neurons has been interpreted as support for motor simulation [23], sometimes referred to as direct matching [24], which proposes that observed actions are mapped onto existing motor schema, supporting both imitation [25] and the understanding of actions [26]. At present simulation theory does not differentiate between object directed and meaningless non-object directed actions, and consequently many studies pertaining to the human mirror system have employed non-object directed action [25], [27]. Thus, according to motor simulation theory [19], the human mirror response should be present for both object directed and meaningless non-object directed actions. Conversely, there is evidence to suggest that the motoric responses to perception are experience dependant [28], [29] and thus may be present only in a subset of actions.

In the current study, we address this issue by comparing whether there is a human mirror response (motor and visual response) to meaningless non-object directed action of the upper limb using two different types of analysis. We aimed to address this issue by looking at a broad scale system, using a conventional subtraction analyses. In addition, we also looked for more fine-grained spatial pattern of activation common to observation and execution of an action. Functional MRI data cannot resolve the presence of single human mirror neurons, as activity that is apparently common to action observation and execution, may originate in closely adjacent but distinct neural populations [30]. Nevertheless, we can investigate the spatial pattern of activity in unsmoothed single subject fMRI data using multivoxel pattern analysis techniques. This approach maximises the spatial resolution of the technique, and reduces inaccuracies produced by warping fMRI images into standard space. Previous similar approaches have produced ambiguous results [31], [32], possibly due to the use of different types of actions and modalities (auditory and visual). In accordance with previous studies of the human mirror system we restricted our stimuli to one type of action per condition [27].

## Methods

### Subjects

Twenty two healthy right-handed subjects (mean age 27 years, range 21 to 32, eight female) participated in this study. All gave informed written consent according to the guidelines approved by Hammersmith Hospital Ethics Committee who provided local ethics approval for this study.

### Stimuli

In the scanner, subjects were able to see a restricted area using a mirror mounted on the head coil. One general confound to studies of the mirror system is that upper limb actions are normally controlled under visual guidance, and separating observation from execution under these circumstances is artificial. In the present study, actions were carried out within this visual field. Subjects were cued either to carry out an action or observe the experimenter carrying out an action. There were two types of action; a transitive actions, in this case object directed action (a pen grasp), and an intransitive action which was a non-object directed action (a horizontal circular hand motion). A restricted set of only two actions was used as macaque studies have demonstrated that mirror neurones are highly specific to the type of action and the goal of an action [19]. Gallese et al., (1996) report that mirror neuron responses are highly stable and do not habituate to repeated presentation of a stimulus, hence we did not foresee any issues of habituation. The experimenter performed the same actions within the visual field of the subject as mirror neurones are known to respond better to real actions, as opposed to video taped actions [12] and respond differently to actions carried out in peripersonal compared to extrapersonal space [33]. There were two baseline conditions, observing a static object (a pen) and observing the background alone. We used live actions, performed within the peripersonal space of the subjects. A raised platform was place on the lap of the subject, angled toward them. An object was placed on this platform in the object directed action conditions. They were given plenty of practice in order to ensure that they could easily carry out these actions however participants did not find this difficult to do.

### Scanning

A 3T Philips system (Intera) was used to acquire 226 T_2_*-weighted echo-planer images (EPI) data (2.2×2.2×2.75 mm3, TR/TE/flip 3000 ms/30 ms/90°) BOLD contrast. An eight-channel array coil and SENSE factor 2 were used as well as second-order shims. The first five volumes were discarded in order to remove the effect of T_1_ equilibration. A T_2_ anatomical volume image was also acquired for each subject.

There were six behavioral conditions each repeated four times: observe object directed (Observe Transitive, Observe_Trans_), observe non-object directed action (Observed Intransitive, Observe_Intrans_), execute object directed (Execute Transitive, Execute_Trans_),execute non-object directed action (Execute Intransitive, Execute_Intrans_),observe object (Observe_Object_) and observe background (Observe_Rest_). The executed actions were self-paced and comprised around 7 actions. Each condition lasted for 21 seconds and was separated by a 6 second instruction block. The blocks were organized in a pseudo-random order. The entire task lasted 11 minutes.

### Standard whole-brain group analyses

#### Pre-processing and analyses

Functional data were analyzed using SPM5 (Wellcome Department of Imaging Neuroscience, London, UK) running on Matlab 7.2 (Mathworks Inc, Sherborn, MA). All functional images were realigned to the first volume by six-parameter rigid body spatial transformation. Functional and structural (T_2_-weighted) images were normalized into standard space using the Montreal Neurological Institute (MNI) template. Functional images were coregistered to the T_2_ structural image and smoothed using a full width half maximum of 8 mm Gaussian kernel. The data were high-pass filtered at 128 Hz. First level analysis was carried out using motion parameters as regressors of no interest at the single-subject level. A random-effects model was employed in which the data were thresholded at p<0.005, uncorrected. A cluster threshold of 25 voxels was employed in order to limit type II errors.

Individual contrasts were carried out to investigate the BOLD response to each condition compared to one of the baseline conditions. Conjunction analyses were carried out by inclusive masking (Observe masked inclusively by Execute) allowing visualization of the BOLD response for action execution *and* action observation (masking threshold of p<0.005). Significant BOLD effects from this conjunction analysis were superimposed on a T_2_-weighted image from one of our volunteers normalized to standard space using the Montreal Neurological Institute (MNI) 152 template. Local foci of maximal activation were then identified using cytoarchitechtonic and probabilistic atlases available within SPM5 [34].

### Multivariate pattern analyses

#### Region of interest analysis

Data were processed using the AFNI toolbox [35], FSL [36] and Matlab 7.2 (Mathworks Inc, Sherborn, MA). The only preprocessing step used was motion correction. Subsequently, a general linear model (GLM) was calculated in AFNI using the unsmoothed functional data. The GLM model included separate regressors for action observation and execution for both object directed and non-object directed conditions as well as nuisance variables modelling mean activation, linear and quadratic trends and head movement. The GLM model resulted in spatial maps of t-values which form the basis of the subsequent correlation analyses investigating spatial overlap.

These correlation analyses were run on individually defined regions of interest as follows: first, a broad anatomically-defined mask was created in MNI152 space using the superior parietal and precentral gyral regions defined with the probabilistic Oxford-Harvard cortical atlas. These masks encompassed regions previously reported for mirror systems by other groups [17] and from unpublished work within the group. This mask was then warped into each subject's native space using FLIRT [37]. This anatomical mask was then combined with a functionally defined mask for each subject, encompassing voxels active (uncorrected p<0.05) to each of the four observe and execute conditions. As such, this approach focuses on only those voxels implicated in both observation and execution for both object directed and non-object directed actions.

Across all chosen voxels in each individual's mask, we calculated the spatial correlation of the t-values (following Downing et al., 2007) between action observation and execution. This was done separately for both object directed and non-object directed actions. The resulting r-statistics were then converted to z-statistics using the Fisher Transform [38]. This resulted in two z-statistics for each subject: one summarizing the overlap between observe/execute object directed actions and another summarizing the overlap between observe/execute non-object directed action. These z-statistics were then used across subjects to compare object directed and non-object directed actions.

#### Whole-brain analysis

A variant of the spatial correlation approach was also run on whole-brain data. A spherical searchlight approach was taken that searches for fine-grained spatial overlap within local areas regions [39]. A 3-voxel radius sphere (containing up to 123 voxels) was passed over the whole-brain. Within each sphere, the spatial correlation between the t-values for observe/execute object directed and observe/execute non-object directed actions were calculated. The resulting r-statistic was converted to a z-statistic and assigned to the center voxel. This resulted in a measure of the spatial overlap of the local neighbourhood surrounding each voxel. These maps of z-statistics in native space were then warped into MNI152 space using FLIRT. Finally, a t-test was calculated for each voxel, evaluating whether there was a significant difference across subjects between the z-statistics for observing and executing a object directed versus an non-object directed action. The resulting probability map of object directed relative to non-object directed spatial correlations was thresholded at p<0.05 (FDR correction).

## Results

### Basic contrasts

**Figure 1:**
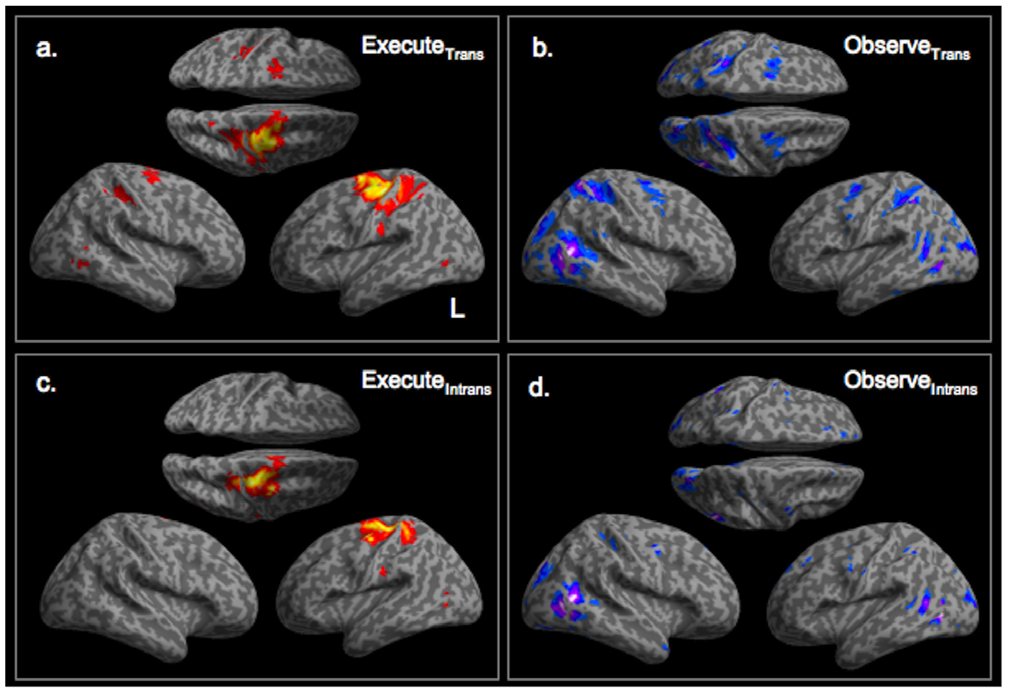
Activity associated with observing and executing actions. BOLD responses associated with observing and executing different types of action compared to a static baseline are displayed in the top panel (p<0.005, cluster threshold = 25). Significant activity associated with execution of an object directed action (Execute_Trans_) are seen in sensorimotor cortices in both hemispheres and right cerebellum (a). Observing an object directed action (Observe_Trans_) was associated with activity in bilateral premotor, superior parietal and lateral occipital areas associated with visual motion (b). Motor responses to execution of a non-object directed action (Execute_Intrans_) are seen in similar regions to the motor responses to Execute_Trans_ (c), however the premotor and parietal activity seen during observation of a object directed action is absent when observing an non-object directed action (d). BOLD responses seen during observing a non-object directed action (Observe_Intrans_) in lateral occipital areas only. All statistical parametric maps displays experimental conditions compared to a passive rest condition (Observe_Rest_) and are thresholded at p<0.005 uncorrected, cluster extent threshold = 25.

Compared to a passive rest condition, executing an object directed action (Execute_Trans_>Observe_Rest_) was associated with significant activity in bilateral sensorimotor cortices, including primary motor, premotor and primary somatosensory cortex, with a greater extent in the contralateral (left) hemisphere (figure 1a, p<0.005, cluster threshold = 25). As expected, observing a transitive, object directed action (Observe_Trans_>Observe_Rest_) was associated with distributed activity in bilateral premotor, superior and inferior parietal cortices and both striate and extrastriate cortex (figure 1b). Execution of a non-object directed movement (Execute_Intrans_>Observe_Rest_) was associated with very similar activity to that of executing an object directed movement (primary somatosensory, motor and premotor cortices) although with a much lesser extent (Figure 1c) and restricted to the contralateral (left) hemisphere. Both execution conditions were also associated with active in lateral occipital cortex in both hemispheres. During observation of a non-object directed movement (Observe_Intrans_>Observe_Rest_), significant BOLD responses were seen in early and late visual cortices, ventral premotor cortex in both hemispheres [BA 6, 48 0 46 and −56 0 36], superior parietal cortex corresponding to area 1 [−58 −8 36], inferior parietal cortex [−34 −46 50] and medial frontal cortex (Figure 1d). Notably, although there were peaks in premotor and parietal cortices during observing a non-object directed hand wave, these activations did not overlap with activations for executing the same movement, the definition of a mirror response.

### Common encoding of action execution and observation in premotor and parietal cortices

**Figure 2:**
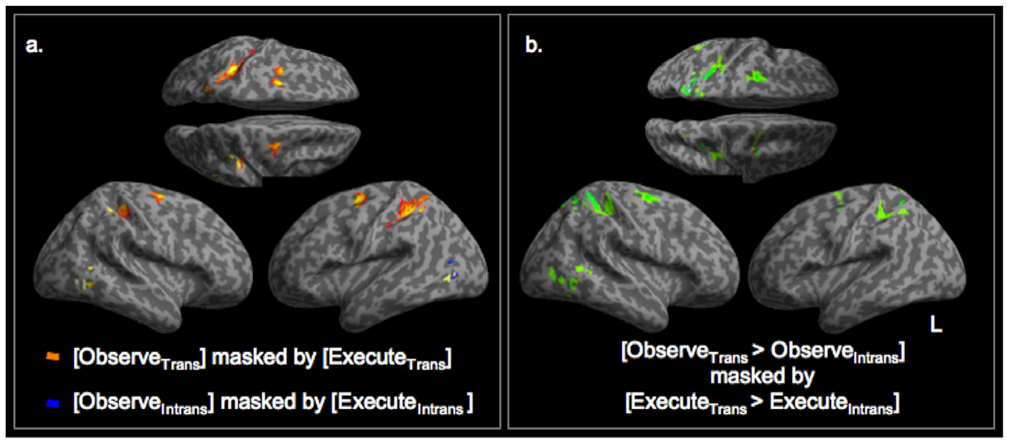
Mirror responses: activity common to execution and observation. Inclusive masking was used in order to look at significant activity common to both execution and observation conditions. BOLD responses to Observe_Trans_+Execute_Trans_ were seen in premotor cortex, dorsal parietal cortex in both hemispheres and right lateral occipital cortex (a, orange). The same approach for Observe_Intrans_+Execute_Intrans_ revealed significant activity in both contrasts in left occipital cortex only (a, blue). A direct comparison of activity common to execution and observation of an object directed actions more than an non-object directed action (Observe_Trans_>Observe_Intrans_)+(Execute_Trans_>Execute_Intrans_) allowed us to highlight voxels that are commonly activated in observing and executing an object-directed grasp more than executing and observing non-object directed movement. This analysis revealed significant activations in bilateral premotor and parietal cortices (b) (28 −48 56, −28 −52 58, 28 −14 56, −30 −4 60, −36 −38 52). The reverse comparison, (Observe_Intrans_>Observe_Trans_)+(Execute_Intrans_>Execute_Trans_), revealed no significant activity.

Before demonstrating the overlap in the fine-grained spatial patterns we performed the standard group-based whole-brain analyses. Inclusive masking of [Observe_Trans_+Execute_Trans_] revealed activity commonly activated during both conditions in premotor (BA 6), primary somatosensory cortex (areas 2 and 3a), inferior parietal cortex (area 2), lateral occipital cortex in both hemispheres corresponding to V5 (Figure 2a, orange) and ipsilateral cerebellum (lobule IV, data not shown on rendered brain image). The same approach applied to [Observe_Intrans_+Execute_Intrans_] revealed no such activity in premotor or parietal areas; the only significant activity in this analysis was in left lateral occipital cortex, corresponding to V5 (Figure 2a, blue). Neither of these approaches directly addresses the issue of mirror responses as they do not control for observing limb movement. In order to do this control for this an inclusive mask of [Observe_Trans_>Observe_Intrans_] and [Execute_Trans_>Execute_Intrans_] was carried out. This approach was designed to search for a mirror response as defined by experiments in non-human primates. This revealed significant activity in bilateral premotor (BA6), areas 2 and 3a of superior parietal cortices in both hemispheres and in right lateral occipital cortex (Figure 2b), lobule VI of the right cerebellum and precuneus (not shown on rendered brain). The reverse contrast [Observe_Intrans_>Observe_Trans_] by [Execute_Intrans_>Execute_Trans_], revealed no significant voxels, even at reduced thresholds. This was due to the fact that the only significant activity that was common to observing and executing a non-object directed movement compared to baseline (not to object directed action) was in left middle temporal gyrus corresponding to V5 (Figure 2a, blue). By investigating the main effects of action observation and action execution we have demonstrated that regions within this network are significantly more active in response to observing and executing an object related action (Observe_Trans_ and Execute_Trans_) than during non-object directed, non-goal directed action (Observe_Intrans_ and Execute_Intrans_) (p<0.005, cluster extend 25, see table 1 for coordinates of peak and subpeaks). A low threshold of p<0.005 uncorrected was deliberately used to reveal the full possible extent of the mirror response, however the mirror response to transitive actions survives correction for multiple comparisons using a false discovery rate of p<0.05.

**Table 1:** Coordinates of from contrasts of interest.

Anatomy	(k)	Z-score		coords
**Contrast: [Observe_Trans_+Execute_Trans_]**				
Inferior temporal gyrus (V5 20%)	114	5.42	R	48 −64 −4
*Middle temporal gyrus*		*4.99*	*R*	*46 −58 2*
Inferior parietal lobe, Area 2 (20%)	539	5.16	L	−34 −44 48
*Superior parietal lobe*		*3.89*	*L*	*−20 −60 52*
*Inferior parietal lobe, Area 2 (20%)*		*3.75*	*L*	*−26 −54 54*
Middle occipital gyrus, V5 (20%)	65	5.06	L	−42 −64 0
Premotor cortex, area 6	259	4.7	L	−24 −6 52
Inferior parietal lobe, Area 2 (20%)	241	4.62	R	36 −42 54
*Post central gyrus, area 2 (60%)*		*4.38*	*R*	*32 −38 48*
Premotor cortex, area 6	115	4.44	R	24 −12 54
Lingual gyrus, area 18	42	3.85	R	10 −66 −8
Cerebellum (VI)		3.46	R	20 −66 −16
**Contrast: [Observe_Intrans_+Execute_Intrans_]**				
Middle occipltal gyrus (V5)	42	5.34	L	−40 −68 4
**Constrast: [Observe_Trans_>Observe_Intrans_]+[Execute_Trans_>Execute_Intrans_]**				
Superior parietal lobe	130	5.76	R	16 −60 60
*Precuneus*		*3.99*	*R*	*8 −58 66*
Post central gyrus, area 2 (30%)	544	5.19	R	26 −48 54
*Superior parietal lobe, area 2 (30%)*		*4.74*	*R*	*30 −50 62*
Post central gyrus, area 2 (80%)		4.22	R	36 −38 56
Superior parietal cortex, area 2 (40%)	290	4.34	L	−28 −50 58
*Post central gyrus, area 2 (50%)*		*3.98*	*L*	*−36 −38 52*
*Post central gyrus, area 3a (10%)*		*3.73*	*L*	*−30 −36 44*
Inferior temporal gyrus, V5 (10%)	255	4.23	R	48 −62 −8
*Middle occipital gyrus*		*4.15*	*R*	*34 −84 0*
*Middle occipital gyrus, V5 (30%)*		*3.87*	*R*	*48 −68 2*
Superior parietal lobe	45	4.22	R	20 −54 52
Precentral gyrus, area 6 (60%)	220	4.18	R	28 −14 56
*Premotor cortex, area 6 (30%)*		*4.09*	*R*	*24 −4 60*
Premotor cortex, area 6 (20%)		2.73	R	36 0 52
Precentral gyrus, area 6 (30%)	167	4	L	−30 −4 60
*Precentral gyrus, area 6 (40%)*		*3.34*	*L*	*−24 −12 52*
Cerebellum (VI)	97	3.88	L	−30 −52 −24
*Cerebellum (VI)*		*3.31*	*L*	*−32 −42 −34*

The coordinates from the contrasts of interest along with the corresponding and z scores are shown in Table 1. Foci of maximal activation were localised using cytoarchitechtonic and probabilistic atlases available within SPM5 (Eickhoff et al., 2005). Coordinates are given in MNI space. Numbers of voxels are listed for main peaks only, not subpeaks.

### Spatial overlap of object and non-object directed action execution and observation

**Figure 3:**
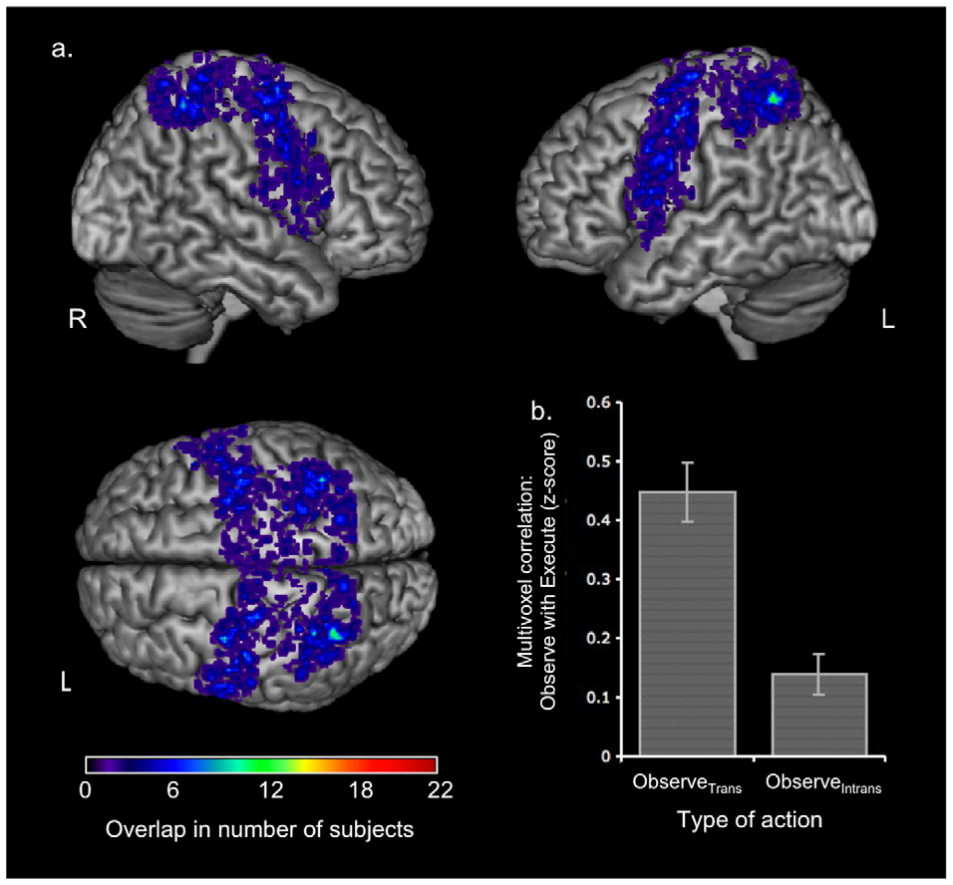
Individual overlaps for Observe and Execute in objected directed and non-object directed action. A mask was used to restrict our analysis to regions significantly active in Observe and Execute conditions (voxels active for all four conditions, p<0,05; within an anatomically defined mask of premotor and parietal regions). These individual masks vary across individuals in widespread premotor and parietal cortices bilaterally (a). Regions of highest overlap are seen in green. The coordinates of peak overlap were −34 −59 64, 36 −42 52, −48 2 35, and −42, −9 58. Within these individual masks, we then looked at the mean correlation between Observe and Execute for the two difference action conditions; object directed and non-object directed. The mean correlation between Observe and Execute was highly significantly greater for object directed action compared to non-object directed action (b).

A mask was created for each subject in their native space restricted to the union of active voxels (p<0.05 uncorrected) in all four action conditions (observe/execute for both object and non-object) within an anatomically defined superior parietal and premotor regions, including those voxels observed in the standard contrasts presented above. Within these premotor and parietal regions, there was a high degree of individual spatial variation in the mirror response (Figure 3a). However, the greatest overlap was in dorsal premotor and parietal cortices in both hemispheres. The peak coordinates for these regions correspond closely with those observed in the whole-brain analysis, but the distribution of all voxels demonstrates the considerable individual variability across subjects.

Overlap in spatial patterns of activation for Execute and Observe for object directed action (Observe_Trans_ and Execute_Trans_) was then compared with those for non-object directed action (Observe_Intrans_ and Execute_Intrans_). The spatial patterns of t-values for observe and execute within this mask were correlated for both object directed and non-object directed action. We found a significantly greater correlation for the object directed action (Figure 3b). The additional control comparison correlating observing a pen with executing a pen grasp did not differ from chance and was significantly smaller than the correlation between Observe_Trans_ and Execute_Trans_.

**Figure 4:**
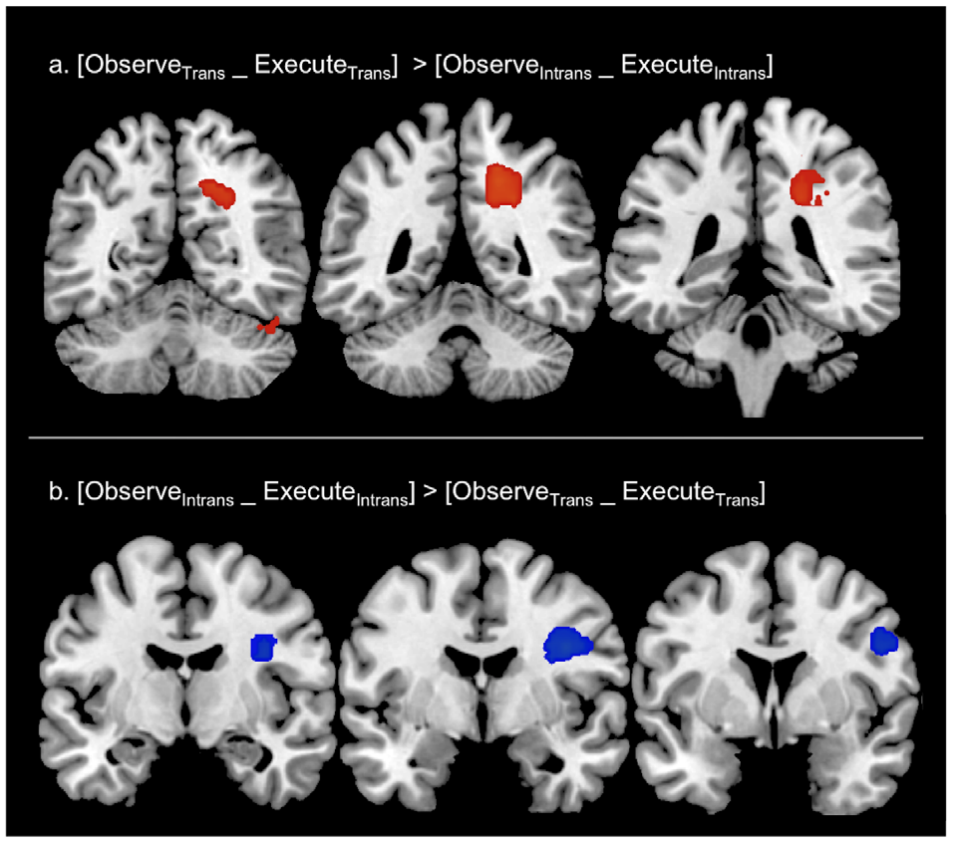
Whole brain analyses of spatial correlation. In addition to the ROI analysis shown in Figure, we also carried out a brain wide search in order to see if there were any other cortical regions displaying a spatial correlation between Execute and Observe conditions. A spherical searchlight was applied to the whole brain and significant correlations were compared for Observe Object directed action and Execute Object directed action (Observe_Trans__Execute_Trans_), and Observe Non-object directed action and Execute Non-object directed action (Observe_Intrans__Execute_Intrans_). Figure (a) shows spatial correlations were greater for Observe_Trans__Execute_Trans_ compared to Observe_Intrans__Execute_Intrans_ in left frontal cortex, inferior frontal gyrus and postcentral gyrus (BA 3). The lower panel shows an anticorrelation in left postcentral sulcus that is present for Observe_Trans__Execute_Trans_ but not for Observe_Intrans__Execute_Intrans_ (b).

These first analyses were restricted to a theoretically determined anatomical region of interest. We therefore wanted to extend this approach to other regions in the cortex that might display correlated spatial patterns but were not significantly activated in the group conjunction. We assessed the whole brain spatial overlap using the spherical searchlight. The resulting map of spatial correlations for Observe_Trans_ and Execute_Trans_, and Observe_Intrans_ and Execute_Intrans_, revealed spatial correlations for object directed and non-object directed actions, respectively, in x, y z (Figure 4).

In this brain-wide analysis, we found significant differences in the correlations between Execute and Observe for object directed compared to non-object directed actions in left post central gyrus (BA 3), inferior frontal gyrus and left frontal cortex (Figure 4) Consistent with the whole-brain activation analysis and region of interest correlation analysis, but at a lower threshold (p<0.001 uncorrected), we also saw correlations in left premotor and parietal areas corresponding to BA 6 and 7 (MNI stereotactic coordinates, −20 −11 63, −20 −73 64, data not shown). These did not survive correction for multiple comparisons, probably because the brain wide approach is using group data and thus incorporates individual variability. A significant anticorrelation was seen in left superior parietal lobe corresponding to Brodmann area 7. This effect was driven by anticorrelated patterns of activity in Observe_Trans_>Execute_Trans_ compared to Observe_Intrans_>Execute_Intrans_.

## Discussion

This study investigated common activity across action execution and observation for both object directed and non-object directed actions. We confirmed the existence of a human mirror response for an object directed action in a network of regions comprising bilateral premotor, parietal cortices, precuneus and extrastriate cortex. This network of regions is similar to the mirror responses found in other fMRI studies for a range of object directed actions including object-directed actions [17] and communicative gestures [22]. However, there was no evidence for a similar mirror response for non-object directed actions. When we considered fine-grained patterns of activation across voxels, we found spatial correlations in premotor and parietal regions, previously reported to be the components of the human mirror network. There was a substantially greater observation/execution overlap in these regions for object directed compared to non-object directed actions. Taken with the results of others [17] these data indicate that the mirror response for object directed actions is not the same as the mirror response for non-object directed actions. This novel finding suggests that the mirror response is not present for all actions and provides evidence for an experience-dependent account of motor simulation.

The striking contrast between the activity seen during a pen grasp and a hand wave highlights the importance of transitivity in action observation. Not only did we see much greater premotor and parietal activity for the object directed action, we also saw much higher correlations for executing and observing the pen grasp. This indicates that if the same populations of cells fire during both observe and execute, these populations are tuned to object directed actions. It is conceivable that the strong correlations observed for object directed actions are driven by the visual processing of an object, which is present in both Observe_Trans_ and Execute_Trans_ but not Observe_Intrans_ or Execute_Intrans_. Neurons that respond to the presence of a three dimensional object are known to reside in area 7 of the parietal cortex [40]. However, if this were the case there would be a correlation between Execute_Trans_ and Observe_Object_, but there was no hint of such a correlation.

The relevance of object directed versus non-object directed actions goes to the heart of what constitutes the mirror system. If the mirror system is a general purpose system for coordinating action and execution with a central role in mediating learning new behaviours and imitation, then the system should not be tied to goal-directed meaningful actions, but should apply to all actions. However, another possibility is that the mirror system does not drive learning, but is an incidental consequence of learning about actions that are both executed and observed. For example, a recent training study has shown temporary novel coupling, revealed by motor evoked potentials, between execution of one action and observation of another [41], [42]. In these circumstances, associative recruitment of the same underlying neural populations would result from Hebbian learning [43], [44]. If this were the case, we might speculate that the greater mirror response observed in this study might be the product of our considerable experience of integrating visual feedback with motor control during object directed versus non-object directed actions. Alternative lines of evidence have also implied that different neural systems may underlie object directed and non-object directed actions. It is known that patients with limb apraxia can suffer from a deficit in object-directed action with preserved non-object directed action and *vice versa* [1]. If mirror neurons were implicated in both object directed and non-object directed actions, the logical conclusion from the clinical observations is that there must be more than one mirror neuron system, but the present experiment provides no evidence that this is the case.

It is important to note that despite the absence of a mirror response for the intransitive action, and the significant difference between the overlap of observation and execution of transitive and intransitive actions, there were clusters of significant activity in premotor and parietal regions during observation of a meaningless intransitive action. These peaks lay in BA6 and both superior and inferior parietal regions supporting previous data [3], [27]. However, there was no overlap between networks for execution and observation of the intransitive action. This empathizes the importance of inclusion of a motor output condition in future studies of the human mirror system and the care that is required in interpreting perceptual responses in these regions as mirror responses [18].

There was an anticorrelation in the comparison of Observe_Trans_ with Execute_Trans_ relative to Observe_Intrans_ compared with Execute_Intrans_. In other words, voxels that were active during the execute condition were not active, or were inhibited, during the observe condition of the object directed action. It has been established that the sensory consequences of internally generated actions are suppressed [45]. Furthermore, a recent study has shown that activity is greater during action observation, and/or below baseline during action execution, in somatosensory cortex [46], [47]. Thus, it is likely that this decorrelated activity relates to the suppression of sensory activity during the Execute condition.

The whole-brain voxelwise activation analysis revealed a strongly bilateral mirror-response for an object directed right limb movement. This is consistent with a recent study that demonstrated that the human mirror response does not lateralize, even when stimuli are presented to one visual hemifield and the response, as in the present study, is unimanual [48]. This has been confirmed by studies using transcranial magnetic stimulation [49], [50]. However, our data from the higher-resolution pattern analysis indicate that spatial correlations show a strong bias to the left hemisphere during observation or execution. This may reflect the fact that there is more actual overlap of the observation and execution neuronal populations in the contralateral hemisphere for an object directed action.

One potential confound in this experiment is that of eye movements. It is possible that there were differences between the extent of eye movements during the object directed and non-object directed conditions, which would result in activity in posterior and frontal eye fields. However are coordinates do not overlap with those reported by studies investigating eye movements [51], [52]. Therefore we do not believe that differences in eye movements can explain these results. Furthermore, a general confound to studies of the mirror system is that upper limb actions are normally controlled under visual guidance, and separating observation from execution under these circumstances is artificial. Thus, in the present experiment, some of the activation in the execution condition may reflect the observation of one's own action. It is likely that the overlapping response in lateral occipital cortex reflected this point. As a result, this study did not provide an exact measure of the human mirror system, but it was designed to demonstrate the maximum possible spatial extent of object directed and meaningless non-object directed mirror systems [18].

In summary, our data indicate that within what is commonly thought of as the human mirror network, there is spatially correlated activity across multiple voxels for observation and execution of actions. This effect is significantly greater for object directed actions. This strengthens the hypothesis that there is common encoding of action execution and observation that arises from the same neuronal populations. The data indicated a clear difference in visuomotor processing of object directed and non-object directed action. These data indicate that there is no single general purpose neural system for matching all observed actions with their motor counterpart, but a system biased for matching observed and executed object directed actions.

## References

[1] Leiguarda RC, Marsden CD (2000). Limb apraxias: higher-order disorders of sensorimotor integration.. Brain.

[2] Rapcsak SZ, Ochipa C, Anderson KC, Poizner H (1995). Progressive ideomotor apraxia: evidence for a selective impairment of the action production system.. Brain Cogn.

[3] Villarreal M, Fridman EA, Amengual A, Falasco G, Gerscovich ER (2008). The neural substrate of gesture recognition.. Neuropsychologia.

[4] Newman-Norlund R, van Schie HT, van Hoek ME, Cuijpers RH, Bekkering H (2010). The role of inferior frontal and parietal areas in differentiating meaningful and meaningless object-directed actions.. Brain Res.

[5] Obayashi S, Suhara T, Kawabe K, Okauchi T, Maeda J (2001). Functional brain mapping of monkey tool use.. Neuroimage.

[6] Obayashi S, Suhara T, Nagai Y, Maeda J, Hihara S (2002). Macaque prefrontal activity associated with extensive tool use.. Neuroreport.

[7] Culham JC, Danckert SL, DeSouza JF, Gati JS, Menon RS (2003). Visually guided grasping produces fMRI activation in dorsal but not ventral stream brain areas.. Exp Brain Res.

[8] Rothi LJ, Ochipa C, Heilman KM (1991). A cognitive neuropsychological model of limb praxis.. Cognitive Neuropsychology.

[9] Pazzaglia M, Smania N, Corato E, Aglioti SM (2008). Neural underpinnings of gesture discrimination in patients with limb apraxia.. J Neurosci.

[10] Pazzaglia M, Pizzamiglio L, Pes E, Aglioti SM (2008). The sound of actions in apraxia.. Curr Biol.

[11] Tkach D, Reimer J, Hatsopoulos NG (2007). Congruent activity during action and action observation in motor cortex.. J Neurosci.

[12] Rizzolatti G, Fadiga L, Gallese V, Fogassi L (1996). Premotor cortex and the recognition of motor actions.. Brain Research Cognitive Brain Research.

[13] di Pellegrino G, Fadiga L, Fogassi L, Gallese V, Rizzolatti G (1992). Understanding motor events: a neurophysiological study.. Experimental Brain Research.

[14] Gallese V, Fadiga L, Fogassi L, Rizzolatti G (1996). Action recognition in the premotor cortex.. Brain.

[15] Ferrari PF, Gallese V, Rizzolatti G, Fogassi L (2003). Mirror neurons responding to the observation of ingestive and communicative mouth actions in the monkey ventral premotor cortex.. Eur J Neurosci.

[16] Fadiga L, Fogassi L, Pavesi G, Rizzolatti G (1995). Motor facilitation during action observation: a magnetic stimulation study.. Journal of Neurophysiology.

[17] Grezes J, Armony JL, Rowe J, Passingham RE (2003). Activations related to “mirror” and “canonical” neurones in the human brain: an fMRI study.. Neuroimage.

[18] Dinstein I, Thomas C, Behrmann M, Heeger DJ (2008). A mirror up to nature.. Curr Biol.

[19] Rizzolatti G, Craighero L (2004). The mirror-neuron system.. Annual Review of Neuroscience.

[20] Buccino G, Binkofski F, Fink GR, Fadiga L, Fogassi L (2001). Action observation activates premotor and parietal areas in a somatotopic manner: an fMRI study.. Eur J Neurosci.

[21] Buccino G, Lui F, Canessa N, Patteri I, Lagravinese G (2004). Neural circuits involved in the recognition of actions performed by nonconspecifics: an FMRI study.. J Cogn Neurosci.

[22] Montgomery KJ, Isenberg N, Haxby JV (2007). Communicative hand gestures and object-directed hand movements activated the mirror neuron system.. Soc Cogn Affect Neurosci.

[23] Gallese V, Keysers C, Rizzolatti G (2004). A unifying view of the basis of social cognition.. Trends in Cognitive Sciences.

[24] Rizzolatti G, Fogassi L, Gallese V (2001). Neurophysiological mechanisms underlying the understanding and imitation of action.. Nat Rev Neurosci.

[25] Iacoboni M, Woods RP, Brass M, Bekkering H, Mazziotta JC (1999). Cortical mechanisms of human imitation.. Science.

[26] Gallese V, Goldman A (1998). Mirror neurons and the simulation theory of mind-reading.. Trends in Cognitive Sciences.

[27] Iacoboni M, Molnar-Szakacs I, Gallese V, Buccino G, Mazziotta JC (2005). Grasping the intentions of others with one's own mirror neuron system.. Public Library of Science Biology.

[28] Hickok G, Hauser M (Mis)understanding mirror neurons.. Curr Biol.

[29] Heyes C (2010). Where do mirror neurons come from?. Neurosci Biobehav Rev.

[30] Downing PE, Wiggett AJ, Peelen MV (2007). Functional magnetic resonance imaging investigation of overlapping lateral occipitotemporal activations using multi-voxel pattern analysis.. J Neurosci.

[31] Dinstein I, Gardner JL, Jazayeri M, Heeger DJ (2008). Executed and observed movements have different distributed representations in human aIPS.. J Neurosci.

[32] Etzel JA, Gazzola V, Keysers C (2008). Testing simulation theory with cross-modal multivariate classification of fMRI data.. PLoS ONE.

[33] Caggiano V, Fogassi L, Rizzolatti G, Thier P, Casile A (2009). Mirror neurons differentially encode the peripersonal and extrapersonal space of monkeys.. Science.

[34] Eickhoff SB, Stephan KE, Mohlberg H, Grefkes C, Fink GR (2005). A new SPM toolbox for combining probabilistic cytoarchitectonic maps and functional imaging data.. Neuroimage.

[35] Cox RW (1996). AFNI: software for analysis and visualization of functional magnetic resonance neuroimages.. Comput Biomed Res.

[36] Smith SM, Jenkinson M, Woolrich MW, Beckmann CF, Behrens TE (2004). Advances in functional and structural MR image analysis and implementation as FSL.. Neuroimage.

[37] Jenkinson M, Smith S (2001). A global optimisation method for robust affine registration of brain images.. Med Image Anal.

[38] Fisher RA (1915). Frequency distribution of the values of the correlation coefficient in samples of an indefinitely large population.. Biometrika.

[39] Kriegeskorte N, Goebel R, Bandettini P (2006). Information-based functional brain mapping.. Proc Natl Acad Sci U S A.

[40] Culham JC, Cavina-Pratesi C, Singhal A (2006). The role of parietal cortex in visuomotor control: what have we learned from neuroimaging?. Neuropsychologia.

[41] Catmur C, Gillmeister H, Bird G, Liepelt R, Brass M (2008). Through the looking glass: counter-mirror activation following incompatible sensorimotor learning.. Eur J Neurosci.

[42] Catmur C, Walsh V, Heyes C (2007). Sensorimotor learning configures the human mirror system.. Curr Biol.

[43] Heyes C (2001). Causes and consequences of imitation.. Trends Cogn Sci.

[44] Keysers C, Perrett DI (2004). Demystifying social cognition: a Hebbian perspective.. Trends Cogn Sci.

[45] Blakemore SJ, Wolpert DM, Frith CD (1999). The cerebellum contributes to somatosensory cortical activity during self-produced tactile stimulation.. Neuroimage.

[46] Rossi S, Tecchio F, Pasqualetti P, Ulivelli M, Pizzella V (2002). Somatosensory processing during movement observation in humans.. Clin Neurophysiol.

[47] Agnew Z, Wise RJ (2008). Separate areas for mirror responses and agency within the parietal operculum.. J Neurosci.

[48] Aziz-Zadeh L, Koski L, Zaidel E, Mazziotta J, Iacoboni M (2006). Lateralization of the human mirror neuron system.. J Neurosci.

[49] Heiser M, Iacoboni M, Maeda F, Marcus J, Mazziotta JC (2003). The essential role of Broca's area in imitation.. Eur J Neurosci.

[50] Aziz-Zadeh L, Maeda F, Zaidel E, Mazziotta J, Iacoboni M (2002). Lateralization in motor facilitation during action observation: a TMS study.. Exp Brain Res.

[51] Konen CS, Kleiser R, Wittsack HJ, Bremmer F, Seitz RJ (2004). The encoding of saccadic eye movements within human posterior parietal cortex.. Neuroimage.

[52] Cornelissen FW, Kimmig H, Schira M, Rutschmann RM, Maguire RP (2002). Event-related fMRI responses in the human frontal eye fields in a randomized pro- and antisaccade task.. Experimental Brain Research.

